# Probiotics, Prebiotics, and Synbiotics: Implications and Beneficial Effects against Irritable Bowel Syndrome

**DOI:** 10.3390/nu13062112

**Published:** 2021-06-20

**Authors:** Elemer Simon, Lavinia Florina Călinoiu, Laura Mitrea, Dan Cristian Vodnar

**Affiliations:** 1 Faculty of Food Science and Technology, University of Agricultural Sciences and Veterinary Medicine, Calea Mănăştur 3–5, 400372 Cluj-Napoca, Romania; simon.elemer@usamvcluj.ro (E.S.); lavinia.calinoiu@usamvcluj.ro (L.F.C.); 2 Institute of Life Sciences, University of Agricultural Sciences and Veterinary Medicine, Calea Mănăştur 3–5, 400372 Cluj-Napoca, Romania; laura.mitrea@usamvcluj.ro

**Keywords:** functional disease, alternative therapy, gut microbiota, gut–brain axis

## Abstract

Irritable bowel syndrome (IBS) is still a common functional gastrointestinal disease that presents chronic abdominal symptoms but with a pathophysiology that is not yet fully elucidated. Moreover, the use of the synergistic combination of prebiotics and probiotics, known as synbiotics, for IBS therapy is still in the early stages. Advancements in technology led to determining the important role played by probiotics in IBS, whereas the present paper focuses on the detailed review of the various pathophysiologic mechanisms of action of probiotics, prebiotics, and synbiotics via multidisciplinary domains involving the gastroenterology (microbiota modulation, alteration of gut barrier function, visceral hypersensitivity, and gastrointestinal dysmotility) immunology (intestinal immunological modulation), and neurology (microbiota–gut–brain axis communication and co-morbidities) in mitigating the symptoms of IBS. In addition, this review synthesizes literature about the mechanisms involved in the beneficial effects of prebiotics and synbiotics for patients with IBS, discussing clinical studies testing the efficiency and outcomes of synbiotics used as therapy for IBS.

## 1. Introduction

Irritable bowel syndrome (IBS) is a functional gastrointestinal disease (FGID) that has no clearly defined causes [1]. Epidemiologic and demographic studies that investigated IBS suggest a heterogeneous worldwide prevalence of the disease that averages around 11% of the population [2], of which 55.0% (95% CI, 46.2–69.4%) of patients were women, and that the average age of IBS patients was 40 (95% CI, 31.2–50.0) years [3].

IBS is a functional disorder associated with the sociologic, economic, and psychological burden and with a low quality of life (QOL), which can be comparable with suffering from anxiety or depression [4,5,6]. Additionally, the indirect and direct costs of IBS are estimated to be around $20 billion per year in USA and up to €3000 annually per capita in European countries with universal healthcare coverage, being comparable with other high-prevalence chronic conditions such as diabetes, persistent asthma or obstructive pulmonary disease [7,8].

IBS is characterized by chronic abdominal pain and altered abdominal habits, like constipation, diarrhea, or alternating constipation and diarrhea, which are also coupled with abdominal bloating, but this association is not applicable for every case of IBS [9,10]. Besides, recent studies showed that IBS influences the gut–brain axis, which links mental health symptoms like depression and anxiety to the disease, making its diagnosis and treatment more difficult [11,12], whereas brain structural alterations were associated with subgroups of IBS subjects underlying the involvement of specific microbes and their predicted metabolites in this disease [13]. The pathophysiology of IBS indicates gastrointestinal (GI) motility dysfunctions, malabsorption of bile acid, and gut microbiota and enteric nervous system alterations. In addition, some studies tightly correlated the IBS with chronic micro-inflammation (also known as low-grade inflammation) at the intestinal mucosa level that induce modifications in the natural GI functions [14,15,16]. This aspect makes the IBS multifactorial nature an impediment in finding an overall efficient treatment. The lack of an efficient IBS treatment, as well as the high diversity of the interventions tested, strongly suggest that the pathophysiology of the disease is not yet fully elucidated [17,18].

The recent studies and clinical evidences underline the importance of the gut microbiota in the pathophysiology of IBS, and led to the use of new types of therapy, such as prebiotics, probiotics, synbiotic, and fecal microbiota transfer (FMT) aiming to modulate the gut microbiota towards a beneficial composition for the IBS patients [19,20,21,22,23,24].

Based on the recent knowledge regarding the efficiency of prebiotics and probiotics use as an alternative therapy for IBS, the purpose of this review is to investigate the latest reported beneficial effects of their combined use as synbiotics in the treatment of IBS in clinical studies, while defining the individual implications of prebiotics and probiotics at the human gut level. Therefore, a comprehensive literature review focusing on the various pathophysiologic mechanisms of action of probiotics, prebiotics, and synbiotics in IBS, including the gut microbiota modulation, the alteration of the gut barrier function, the intestinal immunologic modulation, the visceral hypersensitivity and gastrointestinal dysmotility, and the microbiota–gut–brain communication, was undertaken. The novelty of the present paper is represented by the new interpretation via the multidisciplinary approach of the probiotics in IBS to move forward the knowledge in the field.

## 2. Effects of Probiotics in IBS Pathophysiology

**Figure 1:**
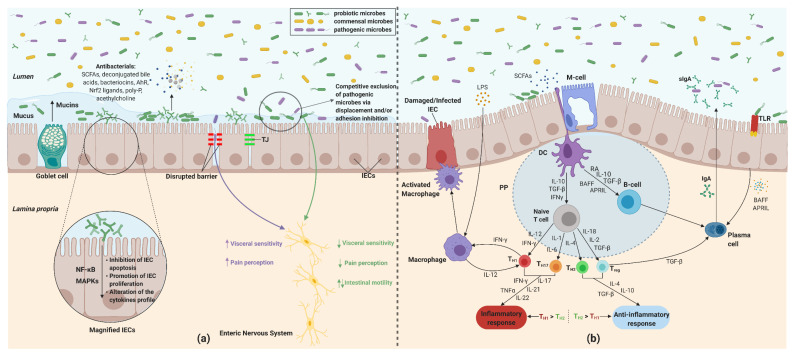
Effects of probiotics at the gut level. In the first half of this image (**a**) is an illustration of the gut microbiota modulation mechanisms of probiotics and their impact on the IECs and the enteric nervous system. Probiotics can alter the gut microbiota via competitive adhesion and/or exclusion of pathogens, production of antibacterial substances such as SCFAs, bacteriocins, AhR, Nrf2 ligands, poly-P, and by stimulating the production of mucins. Additionally, probiotics stimulate the proliferation of IECs, inhibit their apoptosis, alter their cytokines profile through MAPK and NF-κB signaling, and promote the maintenance of tight junctions. Interaction of probiotics with the enteric nervous system leads to a reduction of visceral sensitivity and pain, and modulation of the gut motility. In the second half (**b**) are illustrated the mechanisms of probiotics for immune and inflammatory modulation. The main probiotic-mediated immunologic alteration is realized by their interaction with the DCs, leading to T cells differentiation and stimulation of cytokines production by the immune cells, also of sIgA by the plasma cells. Change of the pro- and anti-inflammatory cytokines profile and of the Th1 to Th2 ratio, due to probiotic interaction, allows them to manipulate the inflammatory response. Abbreviations. SCFA: Short-chained fatty acid; AhR: Aryl hydrocarbon receptor; Nrf2: Nuclear factor erythroid 2-related factor 2; poly-P: Polyphosphate; IEC: Intestinal epithelial cells; MAPK: Mitogen-activated protein kinase; NF-κB: Nuclear transcription factor κB; DC: Dendritic cell; sIgA: Secretory immunoglobulin A; Treg: Regulatory T cell; Th: Helper T cell; TJ: Tight junction; PP: Peyer’s patch; TLR: Toll-like receptor; LPS: Lipopolysaccharide; IL: Interleukin; IFN-γ: Interferon-γ; TGF-β: Transforming growth factor β; TNFα: Tumor necrosis factor-α; BAFF: B-cell activating factor; APRIL: A proliferation-inducing ligand; RA: Retinoic acid. Figure created with BioRender.com.

Probiotics, defined in 2001 by the Food and Agriculture Organization (FAO) and World Health Organization (WHO) as “live microorganisms which when administered in adequate amounts confer a health benefit on the host” [25], present multiple beneficial physiological effects at the gut level, which capacitates their application as IBS therapy. The mechanisms of action of probiotics at the gut level, debated in the next chapters, are shown in Figure 1 and include gut microbiota modulation by competition and inhibition of pathogens adhesion to the gut epithelia by the production of bacteriocins, SCFAs, and biosurfactants; improvement in the gut barrier function of the gut mucosa by downregulation of low-grade mucosal immune activation, increasing the mucus layer, and production of proteins of tight junctions; anti-inflammatory effects via suppression of proinflammatory cytokines; improvement of the gut immunity by stimulating secretory IgA production and enhancement of gut–brain communication [26,27].

### 2.1. Modulation of Gut Microbiota

The influence and importance of the gut microbiota in the health of the host became increasingly clear as imbalances or aberrations of microbiota, also known as dysbiosis, have been demonstrated to play an important role in FGIDs and allergies like infectious and antibiotic-associated diarrhea, food allergy, atopic eczema, inflammatory bowel syndrome, and IBS [28].

The mechanisms of probiotics involved in the modulation of gut microbiota rely on the ability of the probiotic strains or combinations of probiotic strains to inhibit, displace, or interfere in the process of adhesion of pathogenic strains [29,30,31]. However, this is only one of the mechanisms since other mechanisms like bacteriocins are discussed later in the text. Strain, species, and genus variability play an important role in determining the level of adhesion and adhesion competing properties of the probiotics. Several studies showed the ability of specific probiotic strains to displace and competitively inhibit the adhesion of pathogenic strains like *Clostridium difficile*, *Salmonella*, *Escherichia coli*, *Listeria monocytogenes*, *Staphylococcus aureus*, and *Bacteroides vulgatus* [26,29,30,32,33]. The attachment inhibition of pathogenic bacteria by probiotic strains was observed to be realized via steric hindrance at the level of pathogen’s enteric receptors, competitive exclusion of nutrients and mucosal adhesion sites, and by promoting intestinal mucins alterations [34,35].

The production of different antimicrobial substances like bacteriocins, SCFAs, and de-conjugated bile acids is another important mechanism of probiotics involved in modulation of the gut microbiota. SCFAs like butyric, propionic, lactic, and acetic acids are a part of the compounds resulted after the metabolism of carbohydrates by probiotic bacteria and they lower the overall pH of the small intestine, inhibiting the growth of pathogenic bacteria [26,27,36]. Acetic and lactic acids are the most potent organic acids produced by probiotics, exhibiting a strong inhibitory activity against many types of food spoilage bacteria, that include the gram-negative species from *Enterobacteriaceae* and *Pseudomonadaceae* families, lowering the intracellular pH and by the accumulation of the ionized form of the organic acids in the pathogenic cells, which eventually leads to their death [37,38,39]. Additionally, antifungal activity has been reported by Sjögren [40], which found out that saturated 3-hydroxy fatty acids produced by *L. plantarum* MiLAB 14 inhibit the growth of the molds *Aspergillus fumigatus*, *Aspergillus nidulans*, *Penicillium roqueforti*, and *Penicillium commune* and the yeasts *Kluyveromyces marxianus*, *Pichia anomala*, and *Rhodotorula mucilaginosa*, with minimum inhibitory concentrations between 10 and 100 µg mL^−1^ [40].

Bacteriocins or antimicrobial peptides are cationic molecules containing 30~60 amino acids, that prevent the proliferation of selected pathogens by acting at their cytoplasmic membrane level [41]. The bactericidal effect exerted by bacteriocins consist mainly in formation of pores in the membrane, which are deleterious to the targeted cells, and inhibition of cellular wall synthesis [42]. Production of bacteriocins is usually dependent on the lactic acid-producing bacteria (LAB) strains, as nisin is obtained from *L. lactis*, plantaricin from *L. plantarum*, lactacin B from *L. acidophilus*, and bifidocin B, which is a unique bacteriocin produced by *B. bifidum* NCFB 1454, their antibacterial spectrum being narrow and limited to closely related bacteria [39,43,44]. The inhibitory effect of some bacteriocins produced by *L. plantarum* and *L. acidophilus* has been confirmed against several uropathogens [45], rotaviruses, *Helicobacter*, multidrug-resistant *Shigella* spp., *C. difficile*, and in certain conditions against *E. coli* [46].

Probiotic bacteria are also able to synthesize derivatives of the bile acids, known as de-conjugated bile acids, that exhibit a stronger antimicrobial activity in comparison with their bile salts, while the probiotic cell’s self-defense mechanism against these compounds is not yet fully understood [34,47].

### 2.2. Alterations in the Gut Barrier Function

The gut barrier is a defensive mechanism with the function of organism protection from the environment and epithelial integrity maintaining, constantly communicating with the gut microflora and the luminal matters. Several defense methods are contributing to this mechanism, from which the most important are the epithelial tight junctions, mucous layer, and production of antimicrobial peptides (AMPs). Disruption of this function leads to penetration of submucosa by bacteria and antigens, resulting in different intestinal disorders or diseases [34,47]. The effects of probiotics on the gut barrier have been extensively studied, with positive results regarding the increase in mucin expression and secretion by goblet cells, augmentation of β-defensin expression, and secretion into the mucus by epithelial cells, and enhancement of the tight junction stability [48,49,50,51].

The goblet cells, which are found along the GI tract and on other mucosal surfaces, are responsible for expressing the secretion of rod-shaped mucins that form the mucous layer in the lumen or are localized on the cell’s membrane. These secreted mucins are mainly glycoproteins that have more than 50% (wt/wt) degree of glycosylation [52]. The human organism can express secretion of 18 mucin-type glycoproteins, from which the MUC2 is the predominant glycoprotein secreted at the level of small and large bowel mucus. The cysteine-rich residues form inter- and intramolecular disulfide links constitute the skeletal structure of the mucous layer, while the glycan groups confer proteolytic resistance as well as hydrophilicity to mucins [52,53]. The gel layer offers protection to epithelium from harmful bacteria or antigens, acts as a lubricant improving the gut motility and binds the carbohydrates to the epithelium cell’s surface [54]. The pathogen intestinal bacteria must penetrate the mucous layer in order to reach the intestinal cells during infection, thus the mucous layer represents the first barrier [55,56,57].

Promotion of mucus secretion is one of the probiotics mechanisms that improves the barrier function and the exclusion of pathogens. In vitro studies showed an increase in mucin expression, especially MUC2, MUC3, and 5AC in HT-29, Caco-2 cell lines via administration of different probiotic *Lactobacillus* species, but which were strongly dependent on probiotic adhesion to cell monolayers [58,59]. Another study showed an adhesion independent strain of *L. acidophilus* A4 cell extract that exhibited MUC2 increased expression in HT-29 cells [60]. Additionally, increased expression of MUC2, MUC3, and 5AC was reported in a study that tested the use of VSL#3 probiotic mixture (comprised of four strains of *Lactobacillus*, three species of *Bifidobacterium*, and one strain of *Streptococcus*) on HT-29 cell lines [61]. Even though in vitro studies on rats show promising results of the gut barrier improvement, in vivo studies are less consistent because of their few numbers and taking into account the diverse probiotic survival and gut colonization factors and mechanisms, such as presence of surface proteins with role in facilitating the colonization of the human gut through degradation of the extracellular matrix of cells or by facilitating a close contact with the epithelium, the pilus-type IV mediated host colonization and persistence mechanism, auto-aggregation phenotype via co-aggregation mechanism with pathogens [34].

The intestinal AMPs are separated into two families: defensins and cathelicidins. The group of defensins comprises molecules from several classes that are distributed and regulated variably, including α-defensins that are produced by Paneth cells in the crypts of the small intestine, and β-defensins, which are produced more broadly but predominantly by the epithelial cells from the large intestine. The cathelicidins family is formed by antimicrobial cationic peptides expressed in the stomach, ileum, and in the colon, being involved in host defense against pathogens [62]. hBD-2 is a small (3–5 kDa) cationic peptide, which is secreted by epithelial cells throughout the intestine to prevent the reach of pathogens to the epithelium, exhibiting antimicrobial activity against a wide range of bacteria, fungi or viruses [63,64,65]. A significant increase in hBD-2 expressions and/or secretions in Caco-2 cells were shown by in vitro studies, which tested several *Lactobacillus* species, *E. coli* Nissle 1917, commensal *E. coli* strain DSM 17252 S2 G_2_ (sold as Symbioflor 2), or VSL#3 [65,66]. Furthermore, increased levels of hBD-2 have been detected in feces of healthy humans who received Symbioflor 2 twice daily for three weeks, as opposed to placebo-treated individuals that showed no changes [63].

Other important components of the intestinal barrier are the intracellular bonds which are complexes between neighboring epithelial cells that form a semi-permeable diffusion barrier. The constituents of the intracellular complexes are the adherent junctions, desmosomes, gap junctions and the tight junctions, the latter being the most apical and responsible for controlling the permeability of the paracellular pathway [67]. More than 40 proteins are recognized to form these tight junctions, and can be divided in three functional categories: bridge proteins that form a web between adjacent cell membranes; plaque proteins that attach the bridge proteins to the actin cytoskeleton and dual location proteins that also can act as transcription factors and are not continuously associated with the tight junctions [68].

Some probiotic strains of *B. lactis*, *L. acidophilus*, *L. rhamnosus*, and *L. salivarius* exert beneficial properties (indirectly by pre-treatment, or directly) that help to maintain or enhance the intestinal barrier function. In vitro studies have shown that *L. acidophilus* R0052 and *L. rhamnosus* R0011 strains ameliorate the intestinal permeability induced by pathogenic *E. coli* O157:H7 and *E. coli* O127:H6 [69,70,71], while cell-free supernatants from *B. lactis* 420, *B. lactis* HN019, *L. acidophilus* NCFM, and *L. salivarius* Ls-33 improve the integrity of the tight junctions between intestinal epithelial cells which are not weakened [50,72]. Pre-treatment with probiotic strains of *S. boulardii*, *S. thermophiles*, and *L. acidophilus*, and VSL#3 probiotic combination have been shown in many studies to inhibit the decrease in resistance and alteration of the tight junctions that were caused by stress, infection, or cytokines [49,53,69,73,74]. Direct epithelial barrier function modification has been expressed by *L. acidophilus* and *S. thermophilus* probiotic strains that independently decreased the permeability of HT-29 and Caco-2 cells and increased their trans-epithelial resistance [75]. Opposite responses were obtained using bacterial cultured medium or inactivated (by antibiotics or heat treatment) bacteria, which confirmed that live *S. thermophiles* and *L. acidophilus* bacteria are needed to directly enhance the barrier function.

### 2.3. Intestinal Immunologic Modulation

Probiotics are known to influence the GI tract and the gut-associated lymphoid tissue with several effects on intestinal reaction and immune system, which include stimulating the secretion of tumor necrosis factor-α (TNFα) inhibiting metabolites, such as SCFA (e.g., butyrate, secreted proteins (extra-cellular proteins), indole, extracellular vesicles, and bacteriocins; inhibition of nuclear transcription factor κB (NF-κB) signaling in enterocytes; alteration of T-cells towards Th1 polarization and effects on dendritic cells [76]. In addition, new studies highlighted the probiotics’ potential to stimulate the intestinal immune system through the activation of aryl hydrocarbon receptor (AhR) pathway, which is known as an important inflammation regulator [77,78]. AhR recognizes the environmental small molecules of both xenobiotics, and natural chemical compounds such as tryptophan derivatives (indole and kynurenines) [77,79]. The lactic acid producing bacteria activate the xenobiotic responsive Nrf2 pathway, which is also associated with the cytoprotective responses against environmental cellular stresses and with the proliferative control of gut stem cells [80]. Lactobacilli have made their presence noticed in the stress response (for example starvation, acid or oxidative stresses, virulence, motility or biofilm formation) by the synthesis of a storage compound like the inorganic polymer polyphosphate (poly-P) [81]. Last but not least, some particular strains of lactic acid bacteria are able to generate acetylcholine that directly dampen mucosal intestinal inflammation [82,83].

Bifidobacteria, more specifically *B. longum* subs. *infantis* 35,624, has been described to exert immunoregulatory effects via induction of T-regs and attenuation of NF-κB activation [84]. T-regs induction in humans has been shown by the strain along with reduction of proinflammatory biomarkers in patients with different diseases like chronic fatigue syndrome, psoriasis, ulcerative colitis, or IBS [85]. The ability to operate through the pattern recognition molecules or Toll-like receptors (TLRs), like TLR-2 or TLR-4, is another probiotic mechanisms on epithelial cells, resulting in the production of protective cytokines that enhance the regeneration of epithelial cells and inhibit their apoptosis [86]. Epithelial recovery and apoptosis inhibition have been suggested by a study in which the presence of *L. rhamnosus* GG prevented cytokine-induced epithelial cells apoptosis [87]. Co-culture of probiotic *S. typhimurium* or *S. pullorum* strains with either human or mice colon cells lead to activation of antiapoptotic Akt/protein kinase B and inhibition of proapoptotic p38/mitogen-activated protein kinase by TNFα, interleukin (IL) 1α or interferon-γ (IFNγ). Moreover, the commensal *S. pullorum* bacteria influenced the proliferation of epithelial cells via production of factors, in the gut epithelia, that block the degradation of β-catenin, which is implicated in the control of epithelial growth [88].

#### 2.3.1. Dendritic Cells

The effects of different probiotics have been studied both in vitro and in vivo in various systems such as whole blood, lamina propria isolated and monocyte-derived or bone-marrow derived dendritic cells. In vitro studies portrayed the probiotic combination VSL#3 as an effective inducer of IL-10 in human dendritic cells from both blood and lamina propria [89]. Similar results have been recorded in vivo, with patients suffering from ulcerative colitis which were treated with VSL#3, and registered increased IL-10 and reduced IL-12p40 production by dendritic cells [90]. Increased IL-10 was also detected using ELISA by Drakes et al. [91] in human dendritic cells derived from bone-marrow that were incubated with the probiotic VSL#3 mixture. Therefore, a beneficial probiotic-induced cytokine production was demonstrated. However, increased amounts of IL-12, IL-8, TNFα and IL-6 have been induced via stimulation of purified human monocytes by UV-inactivated gram-negative *E. coli* and *V. parvula*, but were practically unresponsive to *L. plantarum* and *B. adolescentis* [92,93]. These results indicate that when monocytes differentiate into dendritic cells, their ability to respond to different commensal bacteria dramatically changes, and they become unresponsive to probiotic gram-positive bacteria.

#### 2.3.2. Macrophage and Monocytes

The tissue macrophages and blood monocytes are secondary efficient antigens contributors to memory T cells. Increased secretion of IL-10 has been shown in macrophages derived from the inflamed colon that were cultured with *L. plantarum* 299 strain [94]. On the other hand, production of IFNγ, IL-12, IL-18, and NF-κB is promoted by *L. rhamnosus* GG in primary human macrophages [95]. Shida et al. [96] demonstrated that in both TLR-2 deficient and wild-types of macrophages, *L. casei* induces production of high levels of IL-12. Besides, *B. bifidum*, *B. breve*, and *B. infantis* were found, in a study by He et al. [97] to stimulate more IL-10 and less IL-12 and TNFα from a murine macrophage-like cell line than *B. adolescentis*, which stressed the importance and dependency of the resulted effects on the probiotic strain used. DNA derived from the probiotic mixture VSL#3 activated NF-B and induced low levels of IL-6 and IL-12 by bone marrow-derived macrophages compared with immunostimulatory oligonucleotides [90]. Moreover, the probiotic strains like those from *Lactobacillus* genus encode genetic loci that are able to stimulate the cytokine production in mononuclear cells such as cells from the peripheral blood [98]. The immune response of dendritic cells by producing IL-10 and IL-12 was investigated in an in silico study conducted by Meijerink et al. [99], who used 42 individual strains of the probiotic model of *L. plantarum* WCFS1 for identifying the gene loci responsible for the modulation of cytokine production. The results of the study revealed that about eight genes from *L. plantarum* might induce the dendritic cells response in producing IL-10 and IL-12 cytokines [99].

#### 2.3.3. Immunoglobulin A (IgA)

The intestinal mucosa contains most of plasma cells (~80%), and it is the place where production of IgA is higher than the other isotype (up to 60 mg/kg/day), being considered the primary response element of the mucosal immune system against antigenic microbes. Probiotic strains fortify the intestinal boundary through the increase of mucins, tight junction proteins, and Goblet and Paneth cells [100]. Enhancement of secretory IgA levels, during infections, have been reported by different strains of probiotics like *L. casei*, which displayed significant alterations in the numbers of cells that produce IgA+ and IL-6, in the small bowel lamina propria of mice [101]. Stimulation of IgA production in B cells have been reported in many other probiotic strains, also shown by a study where subjects consumed fermented milk that contained *L. acidophilus* La1 and *B. bifidum*, followed by vaccination against *Salmonella typhi*, registering a relevant increase of IgA concentration in serum [102]. IgA promotion by probiotic strains has been recorded in children that received *L. casei* GG while being vaccinated against rotavirus [103], also a strong increase of IgA seroconversion has been showed in children suffering of acute rotavirus-induced diarrhea, which were administered *L. casei* GG, during their remission phase [104,105]. *B. bifidum* also shown significant induction of IgA in intestinal mucosal lymphoid cells of mice, with optimal secretion achieved with probiotics encapsulated in alginate microparticles [106].

#### 2.3.4. Toll-Like Receptors

Regarding probiotics, TLR2 is an important receptor that recognizes peptidoglycan, which is the main component of Gram-positive bacteria, including the *Lactobacillus* genus. Vinderola et al. [107] demonstrated in their study the dependency of *Lactobacillus* strains to TLR2, highlighting the interaction of *L. casei* CRL 431 with the epithelial cells through TLR2, interaction that induced an increase in the number of TLR2. Additionally, TLR2 is an important factor in recognition of bifidobacteria, which was shown by Hoarau et al. [108] to stimulate maturation and extended survival of dendritic cell as well as high IL-10 production by a fermentation product from *B. breve* C50 via the TLR2 pathway. Similarly, Zeuthen et al. [109] concluded that the immune-inhibitory effect of bifidobacteria is dependent on TLR2, by showing that TLR2-/- Dendritic Cells’ produced more IL-12 and less IL-10 in response to bifidobacteria. Furthermore, increased expression of TLR2, TLR4, and TLR9, and improved secretion of TNF-α, IFN-γ, and IL-10 in Peyer’s patches have been recorded after probiotic administration in healthy mice [110].

Another receptor relevant for probiotics is TLR9 that can recognize bacterial DNA, which exerts a signaling pathway for mediation of anti-inflammatory responses by unmethylated DNA fragments containing cytosine phosphate guanosine (CpG) motifs that are released by probiotics [111]. Actually, TRL9 detects unmethylated CpG dinucleotides that are plentiful especially in the prokaryotic DNA from intestinal flora [112]. Anti-inflammatory responses induced by probiotics have been shown to be mediated via the TLR9 signaling pathway, preferentially eliciting Th3/T-reg cells, in a study conducted by Rachmilewtis et al. [113], where intragastric and subcutaneous administration of *E. coli* DH5-α probiotic DNA in mice showed amelioration of dextran sodium sulfate-induced colitis in mice with TLR2 and TLR4 deficiency but with no effect in mice with TLR9 deficiency.

Moreover, another significant role in the mediation of inflammatory response induced by probiotics is played by TLR4, which is shown to lead to increasing expression of TLR2, reduction in own expression, and recruitment of inflammatory cells, with an overall pro-inflammatory effect that has the role to control bacterial replication [110,114]. In this regard, *L. casei* CRL 431 strain has been shown to activate TLR4 and was used as a surveillance mechanism for pathogenic bacteria [110].

#### 2.3.5. Th1/Th2 Cell Differentiation

Modulation of immune response towards anti- or pro-inflammatory effects was shown to be stimulated by the interaction between probiotics and a wide variety of intestinal cells like enterocytes, dendritic cells, Th1, Th2, and T-regs; however, this action seems to be highly strain-dependent [115]. Shifting of the Th1/Th2 balance towards anti-inflammatory (inhibition of Th1) or pro-inflammatory responses (stimulation of Th1 generation) has been shown by Shida et al. [116] in food allergy model study in which intraperitoneal injected mice, with heat-killed *L. casei* Shirota, exerted a rise of IL-12 levels in serum, altering the cytokine profile from Th2 to Th1 (pro-inflammatory). Other animal studies support the potential of anti-inflammatory effects of *Lactobacillus* strains in rodents with acetic acid- or methotrexate-induced colitis [117,118]. Additionally, probiotics have been shown to stimulate the production of IL-10, a cytokine that mainly acts to inhibit the inflammatory response [119], effects that were also assessed in human clinical studies that show an increase in production of IL-10, in the serum of children with acute diarrhea, by consumption of *L*. *rhamnosus* GG [120].

Moreover, studies on in vitro cell models like HT-29, Caco-2 cells, enterocyte models showed that probiotics can also modulate the inflammatory effects via the alteration of cytokines produced by intestinal antigen-presenting cells, triggering the orientation of the adaptive response. In this regard, Haller et al. [121] demonstrated a pro-inflammatory effect via expression of IL-1β, IL-8, and TNFα by *L. sakei* in Caco-2 cells, as well as an anti-inflammatory effect caused by *L. johnsonii*, which induced the production of TGF-β in the same cell types. From all the studies that assessed the immunologic modifications caused by probiotics in IBS (and considering also the clinical studies detailed in Table 1 below) we can propose a higher beneficial effect by the consortia (multi-strains) probiotics, precisely *Lactobacillus* and *Bifidobacterium*, whereas there is a high dependency of the effects to strain or strains of probiotics used.

### 2.4. Visceral Hypersensitivity and Gastrointestinal Dysmotility

#### 2.4.1. Probiotics and Visceral Hypersensitivity

Visceral hypersensitivity (VH) is a symptom characterized by urgency for bowel movements, bloating, and abdominal pain that in animals develops in the absence of gut bacteria (germ-free conditions), which then are later normalized by bacterial colonization, concluding the dependency of the nociceptive system’s normal development to the introduction of gut microbes [132]. VH is also associated with the micro-inflammation of the intestinal mucosal, where the infiltrates abound in mast cells, eosinophils, and intraepithelial lymphocytes. Supplementary, mast cells are known to have the ability to induce epithelial and neuro-muscular dysfunctions, and at the same time they can enhance VH and disturbed motility patterns in most FGIDs, postoperative ileus, food allergy, and also IBD [133,134]. In IBS of patients, VH is recognized as one of the diagnostics for pain associated responses, also being known that IBS patients exhibit low tolerance towards rectal distention [135]. Moreover, VH is an important pathophysiological aspect in IBS that may be involved in the disruption of a wide variety of processes such as immune, neural, endocrine, and metabolic processes [136]. One of the immune pathways triggering factors for VH is represented by intestinal permeability, which is a prevalent feature of IBS that correlates with the severity of the symptoms [137].

The positive influence of probiotics on the tight junction proteins between epithelial cells was shown by Hyland et al. [138] to influence intestinal permeability. In the same regard, reduced intestinal permeability was reported in patients suffering from IBS-D (IBS with diarrhea) that were administered multi-strain probiotics containing *S. thermophilus*, *L. bulgaricus*, *L. acidophilus*, and *B. Longum* [139]. More interestingly, an aberrant pro-inflammatory ratio of IL-10/IL-12 was found in IBS patient, being then normalized alongside with symptom alleviation via consumption of the probiotic *B. infantis* 35624 [140]. In addition, under experimental conditions, *Lactobacillus* strains exerted potential to modulate visceral hyper-sensation and to alleviate the visceral pain responses in animal studies (mice and rats) via increase of enterocyte opioid and cannabinoid receptors expression and inhibition of sodium channels [141,142]. Moreover, studies on healthy mice, that had bacterial microbiota disturbed by antibiotics, showed inhibition of VH associated with inflammation after administration of *L. paracasei*, also a clear anti-inflammatory response, and inhibition of afferent pain pathways mark [143].

#### 2.4.2. GI Dysmotility

The GI dysmotility is a symptom frequently found in IBS suffering patients, being more salient in individuals with IBS-D and IBS-C, in which it manifests through delayed transit (IBS-C), respectively through accelerated transit (IBS-D). In this regard, greater emphasis has been shown towards TLRs, which control gastrointestinal homeostasis and are involved in innate immunity, pain modulation, and gastrointestinal motility. Additionally, it was shown that certain TLRs such as TLR4 can detect the pathogen-associated molecular patterns (PAMPs) like lipopolysaccharide (LPS), also flagellin detection by TLR5. The LPS are outer membrane components of gram-negative bacteria that are abundant in the colon and studies on animals showed that low concentrations of LPS are vital for maintaining neuronal survival, while high doses exert neuronal toxicity [144,145]. Moreover, significant delay in GI motility has been observed in mice with mutated TLR4 and in TLR4 knock-out mice [144], while increased TLR4 expression has been reported in colonic biopsies of patients with IBS-D, an increase that reached by 15-fold in patients with inflammatory bowel disease (IBD), and was shown to be upregulated by LPS and flagellin [145,146].

In contrast, probiotics were proved to modulate the gut motility by stimulating the epithelial cells or through direct action over the enteric nervous system. The interaction between the probiotics and the enteric nervous system is known to diminish the diarrheal symptoms from both secretory and infectious diarrhea [147]. Experimental studies have shown that unidentified *Lactobacillus*-based fermentation-product and blocking of calcium-dependent potassium channels by *Bifidobacterium* have managed to suppress post-infective intestinal hypercontractility [148,149]. In addition, in two in vitro studies, human colonic motility was reported as improved by the exposure to the supernatant of *E. coli* Nissle 1917. The same studies indicate that, depending on the dose and period of administration, acute exposure to *L. rhamnosus* GG significantly reduces the acetylcholine-stimulated colonic contractions [150,151]. Moreover, in another experimental trial exerted on rats, the administration of *L. reuteri* led to motility alteration in an ex vivo colonic segment. This fact is due to the decrease in the contractions amplitudes and the increase in the intraluminal fluid filling pressure thresholds for the stimulation of the pressure pulses [142]. In addition, probiotic mechanism involving serotonergic system modulation was also reported to exert anti-motility effect [152]; therefore, targeting serotonin receptors as well as serotonin uptake mechanisms could play a key role in the advance of effective therapies in visceral sensitivity associated disorders like IBS [153].

### 2.5. Microbiota–Gut–Brain Communication in IBS

**Figure 2:**
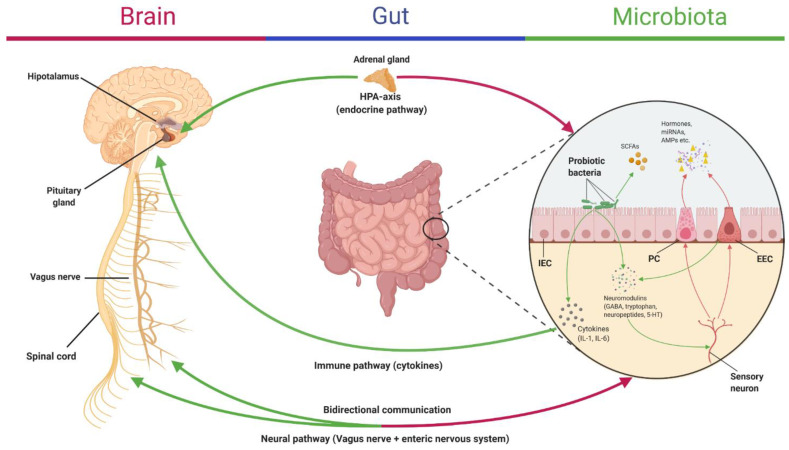
Microbiota–gut–brain axis communication pathways. In this figure, the main bidirectional communication routes between the brain and the gut microbiota are illustrated. The most studied interaction paths between the brain and the gut microbiota are represented by the endocrine pathway, consisting mainly of the HPA axis and enteric endocrine cells (EECs), the neural pathway, that includes the vagal nerve and the enteric nervous system, and the immune pathway, which is mediated via cytokines. Abbreviations. HPA-axis: Hypothalamic–pituitary–adrenal axis; IEC: Intestinal epithelial cell; EEC: Enteric endocrine cell; PC: Paneth cell; SCFA: Short-chained fatty acid; IL: Interleukin; GABA: γ-aminobutyric acid; 5-HT: Serotonin; AMP: Antimicrobial peptide; miRNA: MicroRNA. Figure created with BioRender.com.

The routes of communication between the enteric microbiota and the brain are shown below in Figure 2 and include the vagus nerve, cytokines, gut-secreted neuropeptides, sensory nerves, tryptophan, and SCFA [154], while the opposite route involves the receptor-mediated signaling molecules that are secreted in the lumen mainly by enteroendocrine cells, also recent data indicates that uptake of miRNAs by intestinal microbes can influence their activity [155,156].

Overall results patterns are indicating that fecal and colonic mucosal microbiota are disturbed during IBS and also there is an aberrant host response to the microbiota, thereby leading to altered biological effects on gut function, impaired gut barrier function, immune activation, and communication of intestinal microflora with the central nervous system [157,158]. Small intestinal bacterial overgrowth is a factor thought to have a significant implication in generating the symptoms, or similar symptoms that are common in IBS. However, there is a missing link in the precise differentiation of small intestinal bacterial overgrowth in IBS due to the current limitations of methodologies, such as jejunal aspiration and the breath test [11,159].

On the other hand, the other factors that are complicating the interpretation of IBS are the psychopathological comorbidities that are associated with the syndrome, mainly depression and anxious-like behavior. The experimental models have suggested the influence of gut microflora in behavior, mood, cognition, and neurotransmitting-related functions in animals [160,161,162].

#### Depression and Anxiety as Psychologic Comorbidities Associated with IBS

Depression is a comorbidity that is frequently associated with IBS, more specifically with the elevated levels of inflammation biomarkers such as IL-6, and TNFα. Evidence from rodent studies suggests that stress can produce alterations at the gut barrier level, thereby granting access in the bloodstream to lipopolysaccharides and other molecules that stimulate the secretion of TLRs like TLR4, thus having, as s result, the production of proinflammatory cytokines [34,85,163].

A more recent study, conducted by De Palma et al. [164], underlined the implications of microbiota in the development of behavioral despair using the maternal separation model. Analysis of fecal samples in multiple other studies showed a difference of gut microflora in depressive patients compared with healthy controls, a difference that linked the severity of depression to altered gastrointestinal microbiota [161,165]. The studies also revealed a negative correlation between the severity of depressive symptoms and the *Faecalibacterium* microbes and altered composition of the intestinal microbiota in patients with acute depression [165].

A very important discovery, performed by Sudo et al. [166] in 2004, showed a crucial role of microbiota in the regulation of stress-related responses in physiology, behavior and brain function of animals. In this study, germ-free (GF) mice exhibited exaggerated hypothalamic–pituitary–adrenal (HPA) axis response to stress, modifications that could be reversed by gut colonization with a specific *Bifidobacterium* strain. It must be specified that the reversal effect of colonization on the HPA axis was observed only until a specific age of animals, which implies the likely involvement of brain plasticity. Exaggeration of the HPA axis has also been demonstrated in a later study, in which GF mice exerted abnormal adrenocorticotropic hormone responses to restraint stress, responses that were correlated with a hyperactivity of the HPA axis [167]. Following studies have continued to support the connection between the intestinal microflora and the stress-related responsiveness, reporting that stress exposure in early life or adulthood has the potential to modify the composition of the organism’s microbiota, and contrastingly the microbial populations can modulate the stress responsiveness of the organism [164,168,169,170,171].

## 3. Implications of Prebiotics in IBS

The oldest definition of prebiotics was given in 1995 by Gibson, which defined them as “a non-digestible food ingredient that beneficially affects the host by selectively stimulating the growth and/or activity of one or a limited number of bacteria in the colon” [172]. This definition can still be used nowadays because it encompasses all the required compounds with prebiotic properties. However, a recent agreement of a panel of experts at the International Scientific Association for Probiotics and Prebiotics modified the definition of prebiotics in 2016 to “substrate that is selectively utilized by host microorganisms conferring health benefit” [36]. To summarize, the term prebiotic refers to non-viable dietary substances such as fructan (e.g., inulin), indigestible polysaccharides, galacto-oligosaccharides (GOS), oligosaccharides, or fructo-oligosaccharides (FOS), which preferentially stimulate the growth of a limited number of health-promoting bacteria in the colon and exert health benefits that can include beneficial effects on GI tract, cognitive functions, cardiometabolic health, and bone strength [36].

Owing to the different preferences of the gut microbiota for different energy sources, the diet has the strongest and most direct influence over the microbial profile of the gut microbiota, which are closely related to the species present in the gut [173]. Fermentation of prebiotics leads to the formation of SCFAs like butyrate or propionate which are known for their anti-inflammatory properties [174] and their effectiveness in reducing colitis [175]. The probiotic selectivity towards prebiotics is an important aspect because prebiotics are selectively utilized by certain bacteria in the gut, which mainly includes *Lactobacillus* spp., *Bifidobacterium* spp., *Faecalibacterium prausnitzii*, *Anaerostipes* spp., and *Bilophila* spp., and many other different health-related bacteria that can be promoted through prebiotics. Whereas fibers such as cellulose are poorly fermented in the large intestine, and very few bacteria can utilize it, and pectin, on the other hand, is possibly more specific to bacteria than many currently used prebiotics, including fructooligosacharides [176], and was shown to be a promising prebiotic in promotion of anti-inflammatory bacteria in the colon [177]. The implications of selectivity are clinically important because they mitigate the potential growth of pathogens, or gas-producing microorganisms, such as *Clostridium ssp*., thus preventing the possible unwanted side effects [178]. According to the most recent definition of synbiotic, the probiotic does not necessarily need to have a synergistic effect with the administered probiotic and could be complementary instead [179,180].

Several randomized clinical trials (RCTs) assessed the efficacy of prebiotics in IBS treatment [181]. In 1999, Hunter et al. [182] tested the intake of 6 g/day of oligofructose in 21 patients with IBS but no significant modifications compared with the control group were registered. Another study, conducted in 2000 by Olesen and Gudmand-Hoyer [183], involved 96 patients with IBS that were given 20 g/day of FOS for 12 weeks. An increase in symptoms after four to six weeks suggested that a high dose of prebiotics such as FOS was not only ineffective in IBS symptoms treatment but was potentially dangerous. Negative results were also seen in an RCT in which GOS were randomly administered at 3.5 and 7 g/day to 44 IBS individuals for four weeks. For both doses, a higher predominance of bifidobacteria was found in fecal samples; at the lower dose, improvements in flatulence, bloating and global relief of IBS symptoms were noted, while administration of the high dose of GOS resulted in worsening of the bloating symptoms [184]. A key factor underlined by the results of the RCTs was the dosage; low to moderate dose of prebiotics could provide ameliorative symptoms while a high dosage could amplify bloating in IBS patients [185]. Therefore, considering the adverse effects of a high-fibre diet in patients with IBS, and taking into account the recently published, randomized, controlled study [186,187] where a diet low in FODMAPs (fermentable oligosaccharides, disaccharides, mono- saccharides, and polyols) reduced symptoms in patients with IBS [188], compared with the control group, it is not surprising that a high intake of prebiotics may aggravate the symptoms.

It is important to say that, although a low FODMAP diet is generally successful in reducing IBS symptoms, it does not provide permanent relief, and symptoms usually return after the diet is stopped. Moreover, completely removing fermentable oligosaccharides from the diet could disrupt the entire gut microbial ecosystem, bringing long-term deleterious health effects. Additionally, it has been shown that continuous prebiotic supplementation (GOS) can promote less gas production and more tolerability in vivo [189], and prebiotic supplementation could be better than diet low in FODMAPs in patients with functional gut disorders [190]. Moreover, it should be noted, that prebiotics not do not include only fermentable oligosaccharides which belong to FODMAPs, but also include different types of polysaccharides that were still not tested in individuals with gut disorders. Many polysaccharides (including some insoluble fermentable polysaccharides) have a slow fermentation profile that can render less and delayed gas production and are potentially more tolerable in IBS.

## 4. Synbiotics as Therapy for IBS

The usual term “symbiotic” refers to the combination of prebiotics with probiotics that exhibit a synergistic effect and positively affect the host. The definition of this term was recently updated in May 2019 by a panel of experts in the field (nutritionists, physiologists, and microbiologists) at the International Scientific Association for Probiotics and Prebiotics (ISAPP), who defined synbiotics as “a mixture comprising live microorganisms and substrate(s) selectively utilized by host microorganisms that confers a health benefit on the host” [179]. In accordance with ISAPP, synbiotics are used as nutritional and therapeutic supplements as the synergistic effects of synbiotics consist of the selectivity of prebiotics towards probiotic metabolism, thus ensuring their survival and development in the GI tract [172,179]. According to Kolida and Gibson [191], and to Swanson et al. [179], there are described two general ways that synbiotics can improve the effects of their constituents. Namely, the complementary synbiotics are supplements in which the components are chosen independently, each being responsible for their particular effect, in which case the prebiotic is not necessarily preferentially metabolized by the probiotic strain and could be fermented by the host’s microbiota. In contrast, the other described way is represented by the synergistic synbiotics in which the prebiotics are specifically selected as a substrate for the probiotic strains and are intended to support their growth [179].

Development of synbiotics is reasoned around the sensibility of probiotics towards fluctuations of growth requirements such as pH, oxygen, and temperature, being unable to survive in the human digestive system without the presence of prebiotics [192]. The prebiotics are known to enhance the growth and the metabolic activities of probiotics, as they act as a preservative agent for the live cultures [193,194].

### Clinical Studies

Due to the intestinal effects of probiotics and their synergy with prebiotics, several clinical studies focused on testing the efficiency and outcomes of synbiotics use as therapy for IBS. Although this research is still in the early stages and needing more concluding data with a bigger variety of synbiotics tested, most clinical trials recorded positive results towards improving the IBS symptoms, when they were classified after the evolution chart of IBS diagnosis criteria (Table 2). The majority of clinical studies, which searched the beneficial effects of different synbiotics for patients suffering from IBS, are shown in Table 2, as it reveals significant improvements of IBS markers, with either broad effects like decrease in intensity of bowel habits and abdominal bloating or specific ones such as the increase of SCFAs levels. However, the obtained results show a dependency on the probiotic component of the synbiotic used.

## 5. Discussions

The mechanisms of action of probiotics in IBS, which are diverse, heterogeneous, and strain specific, have been reviewed. The understanding of the mechanism of action of probiotics in IBS should be translated to a more functionally- and clinically-relevant language. Thus, (i) the gut microbiota modulation involves the competitive exclusion mechanism of pathogens by luminal pH, competition for nutritional sources, and production of bacteriocins, SCFAs, and biosurfactants which prevent the proliferation of pathogens and inhibit their adhesion to the gut epithelia. Moreover, (ii) in the gut barrier function, the promotion of mucus secretion (gel layer that offers protection to epithelium from harmful bacteria or antigens by acting as a lubricant improving the gut motility and binding the carbohydrates to the epithelium cell’s surface) is one of the probiotics mechanisms that improves the barrier function and the exclusion of pathogens. Others probiotic mechanisms within the gut barrier function involves the improvement of the integrity of the tight junctions between intestinal epithelial cells and the production of antimicrobial peptides by epithelial cells (cationic peptides) that prevent the reach of pathogens to the epithelium, exhibiting antimicrobial activity. Regarding (iii) the modulation of the immune system, probiotics acts in the differentiation of T-regulatory cells and upregulation of anti-inflammatory cytokines and growth factors, and in the improvement of the gut immunity by stimulating secretory IgA production. At (iv) the gut–brain axis level, probiotics support the regulation of endocrine and neurologic functions for enhancement of gut–brain communication.

There is a rising body of evidence that reveals the modulatory interaction of probiotics and prebiotics with the human intestinal microbiota and its alteration towards a healthier composition for the host. Despite the technological advances in the field of “omics”, their mechanisms of action are not yet fully understood, as their effectiveness in amelioration of IBS symptoms is still debated in the scientific realm [24,198,199]. As concluded in recent studies, the difference of the clinical profiles of IBS patients resulted in different responses following the same probiotic administration, which implies that various factors such as age, gender, diet, bowel habits, the composition of the microbiota, and presence of psychologic comorbidities could influence and be useful predictors of the results in the probiotic interventions [2,24,200]. Regardless of the shortcomings, the research of probiotic and prebiotic use should still be pursued as their combined administration as synbiotics opened a new avenue of study in the field of alternative IBS therapies. With other words, synbiotics constitute a great challenge for the research field, as the synergistic and complementary mechanisms involved in the promotion of the host health are not entirely deciphered [179].

The clinical trials that assessed the effects of synbiotics in IBS patients showed promising results regarding their efficiency in ameliorating IBS symptoms. Although more clinical trials are needed for a more relevant opinion, the current studies show heterogenic beneficial effects. The heterogeneity of the beneficial results varies from amelioration of the overall symptoms to specific symptoms amelioration like increasing of stool frequency, SCFA levels, or reduction of abdominal bloating or abdominal pain. The variability of the beneficial results might be dependent on IBS-related factors such as the subtype, severity of the symptoms, and on the involved probiotic strains, being similar to some of the main recurring factors that could influence the heterogeneity of outcomes of probiotic interventions. However, it is important to underline that, while most probiotics usually have a transitory effect, and are washed out after a person stops consuming them, a synbiotic approach could help with the engraftment of a probiotic as previously demonstrated to be possible in [201].

Therefore, the limitations of prebiotics/synbiotics and difficulties in host engraftment must be addressed as the American College of Gastroenterology (ACG), as of the most recent treatment monograph [202], stated that there is very low evidence for these therapies in IBS, resulting in a weak recommendation for prebiotics and synbiotics. Major reasons were that double-blinded clinical trials were at unclear risk of bias due to failure to report the method used to conceal treatment allocation and significant heterogeneity between studies.

Considering only the probiotics group, the ACG’s stance that probiotics, taken as a group, improve global symptoms, as well as bloating and flatulence in IBS patients, has a low quality of evidence; therefore, results in a weak recommendation. The low quality of evidence results from a significant heterogeneity between studies, evidence of publication bias, or other small-study effects. Moreover, the AGA Clinical Practice Guidelines on the Role of Probiotics in IBS [203] makes no recommendations for the use of probiotics in children and adults with IBS, considering that all the existing studies are marked by: (i) Significant heterogeneity in study design, outcome, and probiotics used; (ii) significant concern for publication bias; and (iii) overall quality of evidence very low. The next-generation probiotics such as *Akkermansia muciniphila*, which is promising in IBS, must be further researched.

## 6. Future Directions

Given the fledgling phase of the use of synbiotics in the field of alternative IBS therapy, as well as the possible dependence of the results of synbiotic administration on their probiotic component, more attention should be allocated towards predetermining the patients’ responsiveness to probiotics. For this, future research should take into account discriminative identification of conclusive probiotic responsiveness markers of IBS patients, like the age group, gender, diet, IBS subtype, bowel habits, presence of psychologic comorbidities, gut microbiota diversity, and composition, that could predict more accurately a positive outcome of the synbiotic intervention. This would lead to a high data input before the clinical intervention, that will offer a better resolution when selecting the probiotic component of the synbiotics. Therefore, among the future directions, the emergent for better quality of research is in need.

## Figures and Tables

**Table 1:** Reported beneficial effects of different synbiotics used as therapy for IBS in clinical studies.

Reference	Study Type	Intervention	Number of Subjects (n)	Subjects Metrics	Inclusion Criteria	Trial Duration	Beneficial Effects
Tsuchiya et al., 2004 [122]	Single-blind, preliminary, controlled trial	10 mL of SCM-III synbiotic consisting of 1.25 × 10^5^ cfu/mL *L. acidophilus,* 1.3 × 10^8^ cfu/mL *L. helveticus,* 4.95 × 10^8^ cfu/mL *Bifidobacterium* and vitamin- and phytoextracts-enriched medium; Complementary/synergistic synbiotic (NS *)	68	20 males, 48 females; mean age, 46 years, range: 36–65 years	Adults with IBS according to Rome II criteria, free from lactose malabsorption, abdominal surgery, overt psychiatric disorders and ongoing psychotropic drug therapy or ethanol abuse	12 weeks	Decrease in intensity of bowel habits and abdominal bloating compared to control group; *p* < 0.01 vs. baseline values and control; <5% reported “not effective” as the final evaluation compared with >40% of patients in the control group.
Colecchia et al., 2006 [123]	Open, uncontrolled, multicenter study	3 g/day synbiotic consisting of *B. longum* W11 and short chain oligosaccharide Fos-Actilight; Complementary/synergistic synbiotic (NS *)	636	250 men, 386 women with age >18 years	Adults diagnosed with constipation-type IBS according to the Rome II criteria	36 days	Increase of stool frequency in patients with IBS-C variant and reduction of abdominal bloating and pain in patients presenting moderate/severe symptoms; ‘‘no symptom’’ class raised from 3% to 26.7% for bloating and from 8.4% to 44.1% for abdominal pain (*p* < 0.0001); for more severe symptoms classes (moderate-severe), symptom frequency decreased from 62.9% to 9.6% and from 38.8% to 4.1% for bloating and abdominal pain, respectively.
Dughera et al., 2007 [124]	Open, uncontrolled, multicenter study	3 g/ day of synbiotic consisting of 5 × 10^9^ cfu/mL *B. longum* W11 Short chain oligosaccharide Fos-Actilight; Complementary/synergistic synbiotic (NS *)	129	NS *	Adults with IBS meeting the Rome II criteria, free from lactose malabsorption, abdominal surgery, overt psychiatric disorders and ongoing psychotropic drug therapy or ethanol abuse	3 months	Increase in stool frequency and lowering of abdominal bloating and pain in patients with moderate and severe symptoms; mean stool frequency before treatment was 12.8 ± 7.1; a significant (*p* < 0.001) increase of movements per month (14.7 ± 8.7 during the first month, 15.8 ± 7.8 during the second month and 16.96 ± 7.8 at the end of treatment) was recorded
Andriulli et al., 2008 [125]	Randomized, double-blind, controlled trial	7 g twice a day of synbiotic Flortec consisting of 5 × 10^9^ cfu/mL *L. paracasei* B21060, 500 mg lutamine, 700 mg xylo-oligosaccharides, and 1243 mg arabinogalactone; Synergistic synbiotic	267	Males and females with age between 18 and 75 years	Adults with IBS meeting the Rome II criteria patients complained about abdominal pain or discomfort as dominant symptom	12 weeks	Decrease of number of bowel movements in comparison with the control group; Number of patients with absent/mild pain increased in the Flortec group (*p* = 0.019)
Min et al., 2012 [126]	Randomized, double-blind, controlled trial	150 mL twice a day of synbiotic product containing *B. animalis subsp. lactis Bb-12* 10^11^ cfu/bottle *S. thermophile* 3 × 10^9^ cfu/bottle *L. acidophilus* 10^9^ cfu/bottle, and acacia dietary fibre; Synergistic synbiotic	130	Males and females with age between 18 and 70 years	Adults with IBS who met the Rome III criteria	8 weeks	Improvement of bowel habit satisfaction in IBS-D predominant patients and overall symptoms in IBS-C predominant patients compared to baseline. Bowel habit satisfaction improved more in the test group compared with the control group (change from baseline of 27.16 vs. 15.51, *p* = 0.010); the improvement in general IBS symptoms was higher in the test group towards the control group (64.2 ± 17.0 vs. 50.4 ± 20.5, *p* < 0.001)
Rogha, Esfahani, and Zargarzadeh, 2014 [127]	Randomized, double-blind, controlled trial	1 tablet three times a day of synbiotic Lactol containing 15 × 10^7^ spores *B. Coagulans* and 100 g fructo-oligosaccharides; Complementary/synergistic synbiotic (NS *)	85	Male and females, mean age: 40 years	Adults with IBS who met the Rome III criteria, predominant symptoms—abdominal pain, diarrhea, constipation	12 weeks	Decrease in abdominal pain frequency (score reduction 4.2 ± 1.8 vs. 1.9 ± 1.5, *p* < 0.001); diarrhea frequency decreased in the synbiotic group but not in the placebo one (score reduction 1.9 ± 1.2 vs. 0.0 ± 0.5, *p* < 0.001); constipation frequency decrease within the two groups (score reduction 0.9 ± 1.2 vs. 0.8 ± 1.1, *p* = 0.561)
Šmid et al., 2016 [128]	Randomized, double-blind, controlled trial	180 g twice a day of LCA synbiotic product consisting of 1.8 × 10^7^ cfu/g *L. acidophilus* La-5 2.5 × 10^7^ cfu/g *B.* BB-12 *S. thermophilus* 2% Beneo dietary fibres; Complementary/synergistic synbiotic (NS *)	76	Males and females with age between 18 and 65 years; test-33 patients, control-43patients	Adults who met the Rome III criteria for a diagnosis of constipation –predominant IBS with symptoms being present for >6 months, and had had symptoms such as abdominal pain, bloating and general digestive discomfort at least twice a week in the last 3 months prior to the clinical trial	12 weeks	No beneficial effects in comparison with placebo group; It was observed an overall improvement of 18% in the total quality of life score from the baseline to the end of the product/placebo consumption period in all the included patients (both control and test groups)
Lee et al., 2019 [129]	Randomized, double-blind, controlled trial	1 capsule of synbiotic (Ultra-Probiotics-500) / day consisting of 10^9^ cfu of each strain (*L. rhamnosus, L. acidophilus, L. casei, L. bulgaricus, L. plantarum, L. salivarius, B. bifidum, B. longum*), 175 mg offructo-oligosaccharides, 150 mg of slippery elm bark powder, 10 mg of herb bennet powder, and 100 g of inulin; Complementary/synergistic synbiotic (NS *)	30	Males and females with age ≥19 years	Adults meeting Rome III criteria for IBS free of IBD, celiac disease, antibiotic treatments, abdominal surgery, pregnancy or breastfeeding, or psychiatric diseases	8 weeks	High doses improved the bowel symptoms and fatigue in comparison with the placebo group. Abdominal discomfort, abdominal bloating, frequency of formed stool, and fatigue were significantly improved in the high-dose group compared with those in the placebo group (*p* = 0.002, 0.003, 0.002, and 0.013, respectively)
Bittner, Croffut, and Stranahan, 2005 [130]	Randomized, double-blind, controlled trial	Once a day one 500 mg capsule of Prescript-Assist^TM^ Safer Medical, Inc., Fort Benton, Montana (Probiotic-Prebiotic complex based on 29 soil microorganisms combined with several substances with leonardite being the predominant component); Complementary/synergistic synbiotic (NS *)	25	23 women, 2 men; age between 20–70 years	Adults with IBS who met Rome II criteria	2 weeks	Reduction in general ill feelings/nausea (reduced by 0.345 standard score units (F (1,46) = 4.26; *p* = 0.042), indigestion/flatulence (reduced by 0.544 standard score units (F (1,46) = 7.83; *p* = 0.008), and colitis (reduced by 0.826 standard score units (F (1,46) = 10.20; *p* = 0.003) compared with placebo group
Moser et al., 2019 [131]	Pilot study	Twice a day of synbiotic mixture (OMNi-BiOTiC Stress Repair) containing 7.5 × 10^9^ cfu of each strain (*L. casei* W56, *L. acidophilus* W22, *L. paracasei* W20, *L. salivarius* W24, *L. plantarum* W62, *L. lactis* W19, *B. lactis* W51, W52, *B. bifidum* W23), Corn starch, Maltodextrin, Inulin, Fructo-oligosaccharides, Potassium chloride, Magnesium sulphate, Manganese sulphate; Complementary/synergistic synbiotic (NS *)	10	Males and females, age between 18–65 years	Adults with IBS symptoms–free of: Chronic inflammatory diseases, immune- or neoplastic diseases, recent application of immune-modifying medication, pregnancy, and alcohol or drug abuse	4 weeks	Increase of SCFA levels, microbial abundance and reduction of symptom severity and fecal zonulin in comparison to baseline (*p* = 0.002). Increased microbial diversity in gastric (*p* = 0.008) and duodenal (*p* = 0.025) mucosal specimens

NS *—not specified.

**Table 2:** Evolution of IBS diagnosis criteria ^1^.

Diagnosis Criteria	Manning Criteria (1978)	Rome Criteria (1992)	Rome II Criteria (1999)	Rome III Criteria (2006)	Rome IV Criteria (2016)
Main diagnosis symptoms	Abdominal pain that is relieved with a bowel movement	Continuous or recurrent symptoms of: abdominal pain, relieved with defecation, and/or disturbed defecation, usually with bloating or feeling of abdominal distension	At least 12 weeks, which need not be consecutive, in the preceding 12 months of abdominal discomfort or pain	* Recurrent abdominal pain or discomfort at least 3 days/month in the last 3 months	* Recurrent abdominal pain on average at least 1 day/week in the last 3 months
Pain and/or defecation associated features	Looser and more frequent stools Sensation of incomplete evacuation Passage of mucus Abdominal distention	Two or more of: Altered stool frequency Altered stool form (hard or loose/watery) Altered stool passage (straining or urgency, feeling of incomplete evacuation) Passage of mucus	At least two of three following features: Relieved with defecation Change in frequency of stool Change in form (appearance) of stool	Two or more of the following: Improvement with defecation Change in the frequency of stool Change in the form (appearance) of stool	Two or more of the following: Related to defecation Change in the frequency of stool Change in the form (appearance) of stool

* These criteria should be fulfilled for the last three months with symptom onset at least six months prior to diagnosis; ^1^ Adapted after: Manning et al., 1978 [195]; Saito et al., 2000 [196]; Sperber et al., 2017 [3]; Lacy et al., 2016 [10]; Lacy and Patel, 2017 [197].

## Data Availability

No new data were created or analyzed in this study. Data sharing is not applicable to this article.
